# Editorial: The Global Challenge Posed by the Multiresistant International Clones of Bacterial Pathogens

**DOI:** 10.3389/fmicb.2017.00817

**Published:** 2017-05-10

**Authors:** Miklos Fuzi

**Affiliations:** Institute of Medical Microbiology, Semmelweis UniversityBudapest, Hungary

**Keywords:** multidrug-resistant pathogen, clone, dissemination, fitness, virulence factor

Multiresistant bacterial pathogens pose a serious problem worldwide making the appropriate treatment of patients with healthcare-associated infections a challenge (www.cdc.gov/drugresistance/pdf/ar-threats-2013-508.pdf).

The spread of antibiotic resistance is either mediated by mobile genetic elements (MGEs) or the dissemination of genetically-related groups of pathogens, “high-risk clonal complexes” (Cantón et al., 2003; Willems et al., 2011; Woodford et al., 2011; Baquero et al., 2015).

Interestingly most multiresistant healthcare-associated bacteria command just a few dominant international clonal complexes causing infections in various geographical areas (Woodford et al., 2011; Willems et al., 2011).

All articles in this Frontiers special issue investigate bacterial species included in the commination list of the Center for Disease Control as pathogens constituting a serious current antibiotic resistance threat (www.cdc.gov/drugresistance/pdf/ar-threats-2013-508.pdf).

It is of utmost importance to identify the determinants associated with and promoting the spread of antibiotic resistance and the dissemination of these multiresistant pathogens. The Topic comprises mostly of population and epidemiological studies investigating antibiotic resistance mechanisms, MGEs and the impact of antibiotic resistance, and the production of virulence factors on the clonal dynamics of a diverse range of bacterial species.

Some papers investigate the production of carbapenemases in *Enterobacteriaceae*.

Lee et al. review the available data on the global spread of KPC-, NDM-, and OXA-48 type carbapenemase-producing *Klebsiella pneumoniae* showing also how major clones disseminated on various continents. The gravity of the situation is emphasized and the remaining therapeutical options explored.

Wang et al. report on the dissemination of the NDM-1 gene in gram-negative pathogens and Sarkar et al. characterized a plasmid carrying *bla*_NDM−1_ from *Salmonella enterica Serovar Senftenberg* a potential vehicle for the spread of the carbapenemase gene.

A number of papers explore clonal dynamics and resistance determinants in non-fermenting bacteria.

Chang et al. showed how carbapenem resistant strains of *Acinetobacter baumannii* clonal complex 92 (international clone II) producing OXA-23 type carbapenemase spread in China at the expense of local isolates.

Farshadzadeh et al. analyzed the clonality of *A. baumannii* strains from burn patients in Iran and genetically characterized the isolates.

Lean et al. compared the genomes of two carbapenem resistant ST195 *A. baumannii* strains showing different susceptibility profiles to polymyxin.

Bahador et al. characterized a novel OXA-23-like gene in *A. baumannii*.

Liu et al. demonstrated that genetic changes and subsequent higher level of expression of OXA-23 β-lactamase resulted in high-level resistance to some β-lactam antibiotics in a strain of international clone II (IC II) *A. baumannii*.

Wong et al. modified the Hodge-test to more reliably detect carbapenemase production in *Pseudomonas aeruginosa* and *A. baumannii*.

Kittinger et al. report on the antibiotic resistance of *Pseudomonas* spp. isolated from various reaches of the river Danube and demonstrate the persistence of a high resistance rate for meropenem.

One paper investigates clonality in *Campylobacter*: Klein-Jöbstl et al. provide data on the clonal affiliation and antibiotic resistance of *Campylobacter jejuni* isolates from Austrian calves.

Some papers report novel findings on the clonality and related resistance mechanisms in *S. aureus*.

Abdulgader et al. provided the first comprehensive review of the clonal division of methicillin-resistant *S. aureus* (MRSA) in Africa.

Shittu et al. report relevant data on the clonal distribution of MRSA in Nigeria. In addition significant associations between various toxin genes and individual clonal complexes are demonstrated.

Sassmansshausen et al. studied the risk factors associated with the carriage of MRSA in healthcare workers in the German part of the Dutch-German EUREGIO and determined the clonal division of the isolates.

Furi et al. investigated how resistance to triclosan develops in *Staphylococci*. Authors elucidated the mechanism of integration for IS1272 which is novel and includes targeting DNA secondary structures. The mechanism and that mediated by related transposons allow for both the clonal and horizontal dissemination of triclosan resistance in *Staphylococci* because it is associated with low fitness cost (Oggioni et al., 2012).

Casarez-Dominguez et al. made a valuable addition to an area still under intense scrutiny: how sub-inhibitory concentration of vancomycin influences the expression of various virulence factors in *S. aureus*.

Two papers investigate the clonal dynamics and related antibiotic resistance mechanisms in *Enterococci*.

Guzman Prieto et al. provide an excellent review of the available data on the emergence of the human clones of *E. faecalis* and *E. faecium* emphasizing the differences between the two species.

Novais et al. used comparative genomics to demonstrate the extensive horizontal dissemination of the PBP5C-type genes conferring reduced susceptibility to ampicillin in *E. faecium* in various sequence types. The work also highlights the co-diversification of PBP5 with the species reflecting the ancestral presence in this background.

Two additional papers investigate relevant antibiotic resistance mechanisms related to clonality in other gram-positive species.

Huang et al. present the first comparative genomic analysis of the conjugative and integrative elements family ICESa2603 in *Streptococci*. The elements are capable of transmitting antibiotic resistance genes to various species and lineages of *Streptococci* since their acquisition does not seem to be associated with any fitness cost.

Kroeger et al. genetically characterized the BC3310 efflux pump—a member of the “unknown major facilitator family 2” (UMF 2)—in *B. cereus*.

The robust impact exerted by fluoroquinolone resistance on the clonal dynamics of various pathogens is explored by two papers.

Chattaway et al. report on the dissemination of fluoroquinolone-resistant non-typhoidal *Salmonella, E. coli*, and *Vibrio cholerae* lineages in Sub-Saharan Africa.

Fuzi demonstrates—referring to data published earlier by his group and by other laboratories—that diverse fitness cost associated with resistance to fluoroquinolones could have substantially contributed to the selection of the major international clones of MRSA, ESBL-producing *K. pneumoniae*, ESBL-producing *E. coli*, and *Clostridium difficile*.

Though, the exploration of the mechanisms governing clonal dynamics and the dissemination of antibiotic resistance will remain a salient issue for a considerable time to come. We believe that the papers published in the Topic have usefully contributed to the better understanding of some of the processes involved and supplement papers investigating the “non-bacterial” constituents of clonal mobility, like proper medical practice and compliance with hygienic standards.

## Author contributions

The author confirms being the sole contributor of this work and approved it for publication.

### Conflict of interest statement

The author declares that the research was conducted in the absence of any commercial or financial relationships that could be construed as a potential conflict of interest.
